# The EMTALA Loophole in Psychiatric Care

**DOI:** 10.5811/westjem.2019.10.45332

**Published:** 2020-01-27

**Authors:** Alexander Schmalz, Nicolas T. Sawyer

**Affiliations:** University of California, Davis, Department of Emergency Medicine, Davis, California

Overhead page: “Code Gray in bed 32.” You find an agitated, combative, and clearly frightened woman in her thirties. After attempting to calm her down and de-escalate the situation, you’re left with no choice but to emergently sedate her to ensure the safety of the patient and your staff. Five security guards hold her down and restrain her to the bed and she’s given intramuscular antipsychotics. When the dust clears, you review her chart and discover she’s been there for 105 hours with diagnoses of schizophrenia with grave disability. This person’s name is Julia and her children and friends visit her regularly. Despite our hopes, the emergency department (ED) is not a place where she can begin to heal. She has been rejected from inpatient hospitalization by 13 psychiatric hospitals in Sacramento and surrounding counties. Julia desperately needs access to psychiatrists and inpatient psychiatric care, and our system is failing her.

Every morning, emergency physicians throughout the state are responsible for reassessing psychiatric patients who are awaiting placement in an acute psychiatric hospital (APH). We’re often greeted by familiar faces, people we’ve met with for three, four, maybe five days in a row. Although these patients have been evaluated and are medically stable for transport, they remain in a persistent state of psychiatric crisis and are stuck in treatment limbo until they receive intensive psychiatric care. As emergency physicians, we are proud to provide an essential service to this highly stigmatized and often marginalized segment of our population, but many of us can’t help but feel an ongoing sense of futility and hopelessness for them. While we were trained in providing the care needed for initial stabilization, we don’t have the skills to meaningfully treat their underlying psychiatric illness. Unfortunately, these patients are trapped in an under-resourced mental healthcare system that is rife with barriers to the intensive treatment they need and deserve.

In an effort to improve access to mental health care, emergency physician and California State Assembly Member Joaquin Arambula, in collaboration with Assembly Member Miguel Santiago, introduced Assembly Bill (AB) 451 in February 2019. The bill would expand the Emergency Medical Treatment and Active Labor Act (EMTALA) to apply to APHs across California, thus subjecting psychiatric care to the same rules and regulations as all other medical specialties that provide coverage for patients in EDs. The hope is that patients with psychiatric disease would be afforded the same access to care as patients with any other disease and we would effectively close “the EMTALA loophole” in psychiatric care. AB 451 seems like a simple, straightforward solution; however, a glance at history and a dive into the current system of care will instill a healthy dose of caution and skepticism.

Mental healthcare in the United States is a patchwork of well-intentioned policies with often wayward results. In 1980, President Jimmy Carter signed into law the Mental Health Systems Act, which aimed to restructure psychiatric care from large, institutionalized asylums with hundreds of beds to a smaller-scale community model. The goal was to make psychiatric care more humane and to safely facilitate reintegration of patients into their communities. In the 1980s, President Ronald Regan ushered through legislation including the Omnibus Budget Reconciliation Act that repealed large portions of the Mental Health Systems Act and slashed federal funding for mental health. These two waves of legislation resulted in the closure of large asylums throughout the country, and then defunded the outpatient mental health treatment network and social safety net that was designed to facilitate a safe and healthy transition for these patients.

There are a few other key regulatory vestiges that shape mental healthcare today. 1988 amendments to the Institution for Mental Diseases (IMD) Exclusion Act barred Medicare from paying for treatment in mental health facilities with more than 16 beds. Put another way, APHs get reimbursed for only 16 patients under their care at any given time and take a financial loss when treating any additional patients. APHs are therefore financially disincentivized to expand the supply of psychiatric care despite our communities’ ever-growing need. An additional rule caps Medicare coverage at 190 total lifetime days of treatment. This is meant to prevent patients from interminably being placed in inpatient psychiatric facilities; however, it serves to arbitrarily limit the potential treatment for patients with the most debilitating psychiatric illnesses. After the 190-day cap is reached, patients are functionally uninsured for the rest of their lives. This is particularly onerous for patients with severe, persistent psychiatric disease who exhaust this paucity of coverage early in life. The IMD exclusion act disincentivizes and stunts expansion of mental healthcare despite immense need.

In 1986, EMTALA was enacted and EDs became the de facto safety net for many patients with mental illness. EMTALA was designed to counteract the growing problem of “patient dumping,” the practice of hospitals refusing to treat people with medical emergencies because of their inability to pay. EMTALA ensured that psychiatric patients had access to physicians; however, it did not ensure timely access to the specialists optimally trained to provide the definitive care needed to treat their illness. While emergency physicians are well versed in preventing self-harm and managing acute psychosis, we are not trained in the behavioral therapy and medication management that can help patients recover from their underlying psychiatric illness.

In 1989, EMTALA was amended to require that hospitals with the specialists needed to stabilize emergency medical conditions accept patients from hospitals without the required specialists. For example, if a patient presents to a small rural critical access hospital with a subdural hematoma, the nearest hospital with an on-call neurosurgeon and open bed is required to accept the patient in transfer. While EMTALA is enforceable by potentially large financial penalties, it is sparingly applied to mental health transfers. In 2012 the California Department of Public Health issued an all-facilities notice that “APHs must provide the care and treatment necessary to relieve or eliminate a psychiatric emergency medical condition within the capability of the facility, including, as necessary, admission or transfer to a psychiatric unit.”^1^ Moreover, the July 2019 Centers for Medicare and Medicaid Services (CMS) State Operations Manual for EMTALA, which contains the regulations and interpretive guidelines states “In the case of psychiatric emergencies, if an individual expressing suicidal or homicidal thoughts or gestures, if determined dangerous to self or others, would be considered to have an emergency medical condition (EMC). Psychiatric patients are considered stable when they are protected and prevented from injuring or harming him/herself or others.”^7^ Unfortunately, public statements from regulatory agencies have largely been ignored. While EMTALA violations related to psychiatric care are vastly under-reported, nearly 20% of all EMTALA fines involve mistreatment of patients with psychiatric emergencies.^2^

The Great Recession of the late 2000s led to additional defunding of mental health systems on the county and state level. In Sacramento, the number of beds at the county mental health facility were halved from approximately 100 to 50 in 2009. This resulted in placement times increasing and patients languishing in local EDs awaiting access to psychiatric care. Health conglomerates such as Sutter, Mercy, and Kaiser responded by reserving beds at APHs in order to move patients with psychiatric needs out of their EDs and free up ED beds for financially profitable medical patients. A tragedy of the commons scenario was created as APHs are paid to reserve beds, but the beds often go unoccupied. The APHs didn’t expand their capacity beyond 16 beds due to the IMD exclusion act, and an already insufficient number of beds became further reduced to protect the monetary interest of large health systems. The bed shortage particularly affects our uninsured and underinsured patients.

The practice of preferentially holding beds for large, private payer groups rather than the patients in most need is morally bankrupt yet ubiquitous. The mechanism APHs use to screen patients before accepting them in transfer is a clear violation of EMTALA standards – every patient being considered for transfer undergoes a “wallet biopsy” as Sacramento APHs require the referring hospital to transmit a face sheet that includes the patient’s insurance status. APHs often deny uninsured, underinsured Medi-Cal patients, or Medicare patients who have exhausted their 190 reimbursement limit based off this information. Patients treated in an ED for an acute mental health condition are particularly vulnerable as 45% of are enrolled in Medi-Cal, 19% have Medicare, 7% are uninsured, and only 25% have private insurance.^3^ This leads to a two-tiered system in which patients with acute, complex psychiatric needs typically board in EDs for days or weeks, while better-funded and less debilitated patients are often placed within hours.

ED boarding is a health risk that disproportionately affects patients with mental health needs. There is a 2.5% mortality rate for patients admitted in less than two hours compared to a rate of 4.5% for patients boarding more than 12 hours.^4^ Prolonged boarding is also associated with delays to pain medication and diagnostic studies, and lower patient satisfaction.^4^ In our ED, the vast majority of patients boarding for more than 12 hours, and nearly all of the patients waiting more than 24 hours, are in psychiatric crisis. This is not an isolated trend. A 2012 study found that psychiatric patients remain in EDs 3.2 times longer than non-psychiatric patients.^5^

Decreased access to psychiatric care is not just an inconvenience. It harms all of our patients, and we applaud Assembly Members Arambula and Santiago for their efforts. Moreover, we would be remiss not to mention the hard work by the California Chapter of the American College of Emergency Physicians for their outstanding advocacy work on this important issue.

We believe that AB 451 will make real change for our patients, but it will take more work to cure our broken system. AB 451 will eliminate the ability of large payer groups to monopolize beds, expand and hasten access to mental healthcare, reduce preferential placement based off payer status, and help ensure that patients in psychiatric crisis get the care they need when they need it. That said, generations of myopic legislation have created a system in which the supply of mental health beds will continue to be outstripped by demand unless we increase funding and build capacity. EDs are the release valve for a mental health system that can’t treat all its patients and the patients boarding in our EDs can be conceptualized as overflow for a system that doesn’t have the capacity to handle the volume of need that exists. We will continue to see patients like Julia flowing into EDs throughout the state until there are more psychiatric providers and psychiatric beds. Patients like Julia need and deserve a well-funded mental healthcare system that can serve every patient with psychiatric needs. It is our responsibility to our patients to continue undoing decades of self-sabotaging policy by increasing funding for mental healthcare, and collaborating with our psychiatry colleagues to grow the capacity of the mental healthcare system so that our patients can get access to the care that they need and deserve.
